# Firefly swarm intelligence based cooperative localization and automatic clustering for indoor FANETs

**DOI:** 10.1371/journal.pone.0282333

**Published:** 2023-03-30

**Authors:** Siji Chen, Bo Jiang, Tao Pang, Hong Xu, Mingke Gao, Yan Ding, Xin Wang

**Affiliations:** 1 School of Computer Science and Technology, Chongqing University of Posts and Telecommunications (CQUPT), Chongqing, China; 2 The 32nd Research Institute of China Electronics Technology Group Corporation (CETC 32), Shanghai, China; 3 School of Communication and Information Engineering, CQUPT, Chongqing, China; Dai Hoc Duy Tan, VIET NAM

## Abstract

At present, the applications of multiple unmanned aerial vehicles (UAVs) are becoming more and more widespread, covering many civil and military fields. When performing tasks, UAVs will form a flying ad hoc network (FANET) to communicate to each other. However, subject to high mobility, dynamic topology, and limited energy of FANETs, maintaining stable communication performance is a challenging task. As a potential solution, the clustering routing algorithm divides the entire network into multiple clusters to achieve strong network performance. Meanwhile, the accurate localization of UAV is also strongly required when FANETs are applied in the indoor scenario. In this paper, we propose a firefly swarm intelligence based cooperative localization (FSICL) and automatic clustering (FSIAC) for FANETs. Firstly, we combine the firefly algorithm (FA) and Chan algorithm to better cooperative locate the UAVs. Secondly, we propose the fitness function consisting of link survival probability, node degree-difference, average distance, and residual energy, and take it as the light intensity of the firefly. Thirdly, the FA is put forward for cluster-head (CH) selection and cluster formation. Simulation results indicate that the proposed FSICL algorithm achieves the higher localization accuracy faster, and the FSIAC algorithm achieves the higher stability of clusters, longer link expiration time (LET), and longer node lifetime, all of which improve the communication performance for indoor FANETs.

## Introduction

Recently, unmanned aerial vehicle (UAV) has attracted growing interests and emerged as the state-of-the-art technology for data collection and been widely deployed in many applications such as the Internet of Things (IoT) and smart cities because of its advantages of fast deployment, high coverage and high mobility [1]. Managing a large number of UAVs to realize a large-scale UAV autonomous swarm, self-organized UAV formation is proposed as a promising network structure, which is also called flying ad hoc networks (FANETs) or UAV ad hoc networks (UANETs) [2–4]. FANETs are special form of mobile ad hoc networks (MANETs) and vehicular ad hoc networks (VANETs), which use UAVs carrying multiple sensors as network nodes to transmit data to each other, and UAVs can negotiate to complete the collaborative operation and build the network independently. FANETs are not limited to the network structure centered on the control base station and can achieve high-speed sharing and autonomy of information due to the rapid deployment, flexible configuration and strong maneuverability of UAVs. FANETs not only have the characteristics of centerless, self-organizing and dynamic topology of mobile self-organizing network, but also face new challenges, such as frequent topology update under high dynamics, limited node energy, and fragile network security [5–8]. In the case of large-scale and high-speed FANETs, ensuring stable communication is a challenging task.

When the UAV swarm performs indoor tasks, such as person rescue, the FANET will be applied in an indoor environment. In this case, the high-precision location information is a mean to support UAV swarm formation, and it is also an important prerequisite for stable UAV swarm communication. Due to the poor indoor performance of global positioning system (GPS), a more accurate indoor localization method is extremely important in the indoor FANET. Many researchers have proposed some improved algorithms [9]. However, these algorithms cannot improve the measurement accuracy by using redundant measurement data, and it is difficult to find the optimal solution. At the same time, they require highly accurate initial solutions, and the performances deteriorate seriously in the case of large measurement noise. The above localization algorithms generally assume that the measurement error follows the Gaussian distribution, therefore the analytical expression of the likelihood function can be obtained, which can be solved by using the maximum likelihood method.

In recent years, the clustering algorithm is a hot research topic in ad hoc networks which divides the entire network into clusters by adopting optimal strategies to achieve stronger network performance. With the help of the good performance of localization, the clustering routing algorithm can be deployed as it is one of the effective methods to eliminate data redundancy, promote data fusion within the network, reduce traffic and communication distance, and save network resources and node energy [10].

The clustering algorithm in the FANET divides the UAVs into several clusters, with each cluster composed of a cluster head (CH) and a number of cluster members (CMs), and an allocation algorithm is needed to assign several finite number of objects to the cluster according to the task [11–13]. In such a configuration, only the CH takes responsibility for intra-cluster and inter-cluster communication in a higher level network, while the CMs in the cluster only need to complete the intra-cluster communication in a lower level network [10]. This hierarchical structure has high scalability and robustness by constructing different level networks. The communication burden of the CHs, however, are heavier since they have to transmit data between clusters and manage changes in CMs [14]. In addition, the unevenly distributed CMs also leads to a decrease in the lifetime and communication stability of the entire network. Therefore, in the process of clustering, CH selection and cluster formation are important tasks for establishing the cluster structure. There were various clustering algorithms proposed in the literature, however, most of them only considered the CH selection and manual clustering, and did not take into account the CM trajectories and automatic clustering.

In order to solve the localization and clustering problems, in this paper, we first propose a firefly swarm intelligence based cooperative localization (FSICL) algorithm which combines the computational ability of the Chan algorithm and the swarm search ability of the firefly algorithm (FA) to cooperative locate UAVs. Secondly, we propose a firefly swarm intelligence based automatic clustering (FSIAC) algorithm which formulates a more appropriate fitness function for CH selection and design the movement mechanism of UAVs for cluster formation. Compared with the existing works, this paper not only considers the localization of UAVs, but also considers the CH selection and flight trajectories of UAVs for building clusters with more stable intra-cluster communication in a 3-D area.

The main contributions of this paper are summarized as follows:

1. Aiming at the problem of inaccurate indoor GPS localization, we combine the computing ability of the Chan algorithm and the swarm search ability of the FA to locate UAVs more accurately. The first solution of location is obtained by the Chan algorithm, the FA then is used to obtain a more accurate solution in the limited cube search zone centered on the first solution;
2. Considering the high mobility, dynamic topology and limited energy of UAVs in FANETs, we formulate a more reasonable fitness function consisting of link survival probability, node degree-difference, average distance and residual energy for the CH selection;
3. Inspired by the social behavior of fireflies, we take the fitness value of the UAV as the light intensity of the firefly, and the UAV with highest light intensity in the cluster is selected as the CH. Then the FA is employed to spontaneously generate optimized clusters for robust transmission which allows CMs adjust their locations on the basis of their fitness values to automatically track the motion of CHs in the UAV swarm, which makes the topology within the cluster more stable;
4. Extensive simulation results verify the network performance gains of the proposed scheme compared with several existing algorithms. Specifically, the proposed FSICL algorithm estimates locations of UAVs more accurate in less time. With the help of the accurate localization, FSIAC algorithm achieves the higher cluster stability, longer LET, and longer node lifetime. All simulation results prove that the proposed FSICL and FSIAC algorithms could be utilized in practice as a new localization and clustering algorithm respectively for indoor FANETs.

The rest of this article is organized as follows. In Section “Related work”, we analyze the related work. In Section “Preliminaries”, we present the system model, FA, UWB localization and Chan algorithm. In Section “Proposed algorithms”, we introduce our proposed FSICL and FSIAC algorithms in detail. In Section “Simulation results”, we present the performance evaluation results for the proposed algorithms. Finally, we conclude the paper in Section “Conclusion”.

## Related work

Ultra wideband (UWB) is one of the most popular technologies adopted for indoor localization. In UWB localization systems, the time difference of arrival (TDOA) is usually used as it does not directly use the arrival time of the signal, but uses the time difference of the signals received by multiple base stations to determine the location of the mobile station. Compared with the time of arrival (TOA) algorithm, significantly fewer communications are required to complete a localization, and the localization accuracy is also improved [15–17].

The core of localization based on TDOA is to solve the nonlinear localization equations and there were three main methods researched, the first is analytical method, such as Fang algorithm [18] and Chan algorithm [19]; the second is iterative method, such as Taylor algorithm [20]; the third is intelligent method, such as particle swarm optimization (PSO) algorithm [21]. Among them, the Fang algorithm obtains the location of the unknown UAV by linearizing the hyperbola with the help of three and only three base stations. The Chan algorithm obtains the location by solving the non-recursive hyperbolic equation, and localization accuracy increases with the increase of base stations. However, for measurements with large errors in the real environment, such as in the presence of non-visual propagation, this algorithm’s performance is significantly degraded. The above two analytical algorithms have the advantages of simple principle and low computational complexity. As an iterative method of Taylor algorithm, it obtains the initial iteration value based on Taylor series expansion to perform least squares estimation, and then the local least squares solution of the location estimation error is solved to update the location of the UAV. The disadvantage of this algorithm is that it is computationally intensive and easily to fall into the local minimum if the initial target location is poorly chosen. Compared with the above two mathematical methods, the PSO algorithm can calculate the location faster and is less affected by noise, but it also faces the problem of easily falling into the local optimum.

Based on the good performance of localization, the clustering routing algorithm can be deployed to better manage the network topology. There were various typical clustering algorithms proposed in the literature, e.g. the lowest-ID clustering algorithm (LIC) [22], the highest-connectivity clustering algorithm (HCC) [23] and the weight-based clustering algorithm (WCA) [24]. In the LIC, each UAV in the network corresponds to a unique ID number, and the UAV with the smallest ID number among the adjacent UAVs will be selected as the CH. Different from the LIC, the HCC is clustered based on the degree of connectivity. By calculating the number of neighbor UAVs, the UAV with the largest number of neighbor UAVs is selected as the CH. In comparison to the LIC and HCC algorithms, the WCA algorithm does not consider a single factor, but integrates multiple factors together, such as node degree, mobility, residual energy, distance from neighbor UAVs. In this way, it can better adapt to different scenarios. An energy and mobility-aware stable and safe clustering (EMASS) algorithm was proposed based on the WCA in [25], which considers three parameters of mobility, stability, and safety to provide stable routing and efficient data collection in FANETs. However, the computation process takes too long and does not take into account the node degree.

Several swarm intelligence and machine learning-based clustering schemes were also proposed in recent years. An improved grey wolf based clustering optimization algorithm (GWCOA) was proposed in [26]. It imitates the leadership hierarchy and hunting mechanism of grey wolves to create efficient clusters. However, the parameter of link quality in the fitness function for CH selection is not considered in this paper. A clustering strategy was presented by [27], which uses the K-means algorithm to quickly cluster the entire network, and then implement a CH selection algorithm based on deep Q-learning (DQN) in each cluster. In this paper, the reinforcement learning is used to adaptively to select the most appropriate CH, but the authors allocates the clusters manually without considering automatic clustering. A bio-inspired mobility-aware clustering (BIMAC) algorithm was proposed in [28], which ameliorates the model of physarum polycephalum for CH selection to adapt to mobile ad hoc networks. The algorithm combines with the mobility characteristics of UAVs, so it can effectively establish and maintain clusters, and improves the average LET and average CH holding time. But the algorithm does not take into account the flight trajectories of UAVs. In [29], an intelligent clustering routing approach (ICRA) algorithm was proposed which takes four factors into account to calculate the utility of a UAV, including the residual energy of the UAV, the degree centrality of the UAV, the velocity similarity, and the link holding time. The reinforcement learning was utilized to help clustering strategy adjustment module learn the benefits brought by adopting different strategies to calculate the UAVs utility in a specific network state and determine the optimal clustering strategy accordingly. However, the authors does not consider the trajectories of CMs, which may cause the UAVs acting as CMs to change too fast and the topology of the intra-cluster to be unstable.

In this paper, inspired by the swarm intelligence algorithms proposed in [30–34], we propose FSICL and FSIAC algorithms to make UAV localization more accurate and communication of UAV swarm more stable. Compared with several algorithms, the proposed algorithms estimates locations of UAVs more accurately, and achieves the higher cluster stability, longer LET, and longer node lifetime in the indoor FANET.

## Preliminaries

### System model

**Fig 1:**
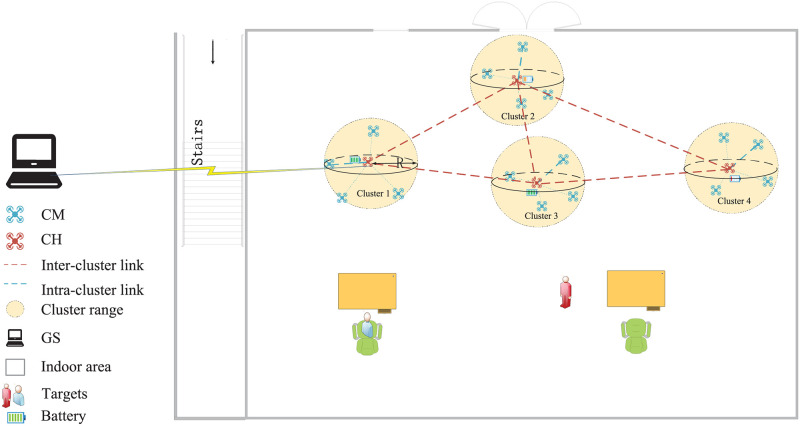
Clustering model in FANETs.

For a scenario of target resuce, we consider a typical network model deployed by *N* small random moving UAVs with different battery power flying at different altitudes and 1 ground station (GS) shown in Fig 1. All UAVs are equipped with long-distance and short-distance wireless communication protocols. To improve the dynamic network performance, the UAVs are organized into several clusters, and each cluster is composed of a CH and CMs. CHs work in 2.4GHz frequency band and are responsible for intra-communication and inter-communication, while CMs work in 5GHz frequency band and only need to complete the intra-cluster communication. The task of the UAV swarm is to search the indoor area and transmit information of the target person back to the GS through single/multi-hop routing on the premise of ensuring the communication quality.

We define that UAVs in the communication range *R* form a cluster. All UAVs are equipped with UWB localization system and aware of locations of themselves and neighbors by sending Hello message to their neighbor UAVs within *R* after every *T* seconds period.

### Firefly algorithm

In our work, the FA is first considered as it is a novel swarm intelligence method. The FA is proposed by [34] as a heuristic algorithm inspired by the blinking behavior of fireflies. The main purpose of a firefly’s flash is to act as a signaling system to attract other fireflies.

The firefly with the higher luciferin is brighter than others, and therefore, has the higher light intensity. Each firefly will move towards a brighter one within its vision range (i.e., communication range) *R*, which is set according to the maximum communication radius of the UAV. Thus, if two fireflies are far apart, the brighter firefly will not attract the darker one, which leads to the fact that the entire swarm can automatically divide into several clusters and the darker fireflies will track the brighter fireflies. It is similar to the case that UAVs need to gather around the CHs to form stable clusters. If the swarm size is much larger than the number of clusters, this enables all UAVs to efficiently find both global and local optimums simultaneously, where the global optimum is the optimum of all the local optimums.

In the simplest case for maximum optimization problems, the light intensity *I* of a firefly at a particular location ***x*** can be chosen as *I*(***x***(*t*)) ∝ *f*(***x***(*t*)), where *f*(⋅) is objective function value at *t* time. Attractiveness *β* of firefly *n* can be defined as:

$$\begin{array}{c} \beta \left(n\right)=\beta_{0}e^{-\mu d_{nm}^{2}} \end{array}$$ (1)

where *d*_*nm*_ is the distance from firefly *n* to firefly *m*, *β*_0_ is the attractiveness at *r* = 0. *μ* is a fixed light absorption coefficient. For instance, the distance between any two fireflies *n* and *m* at their respective locations ***x***_*n*_ = (*x*_*n*_, *y*_*n*_, *z*_*n*_) and ***x***_*m*_ = (*x*_*m*_, *y*_*m*_, *z*_*m*_), is the Cartesian distance:

$$\begin{array}{c} d_{nm}=\|x_{n}-x_{m}\|=\sqrt{{\left(x_{n}-x_{m}\right)}^{2}+{\left(y_{n}-y_{m}\right)}^{2}+{\left(z_{n}-z_{m}\right)}^{2}} \end{array}$$ (2)

where *x*_*n*_, *y*_*n*_, *z*_*n*_ is the 3-D coordinates of the *n*th firefly.

Firefly *n* will move in the direction of other fireflies *m* that are brighter than it:

$$\begin{array}{c} x_{n}=x_{n}+\beta_{0}e^{-\mu d_{nm}^{2}}\left(x_{m}-x_{n}\right)+\lambda \left(\text{rand}-\frac{1}{2}\right) \end{array}$$ (3)

where the second term is due to the attraction while the third term is randomization with λ being the randomization parameter. rand is a random number generator uniformly distributed in [0, 1].

It should be noticed that when *μ* equals 0, the FA is the same as the PSO, and when *μ* equals 1, the FA is the same as random search algorithm. The biggest difference between the FA and PSO is that the FA can find the global optimum and local optimums at the same time, and it is more difficult to fall into the local optimum. In the cluster, each CH represents a local optimum which allows their CMs to automatically follow to perform the task. This mechanism considers the flight trajectories of UAVs and its benefit is that it builds a more stable topology within the cluster and thus achieve the more stable intra-cluster communication. Therefore, the FA with these characteristics is more suitable for simulating the clustering process in FANETs.

### UWB localization based on TDOA

Due to the high data transmission rate (up to 1Gbit/s), strong anti-multipath interference ability, and low power consumption, UWB communication becomes one of the most popular technologies adopted for indoor localization. TDOA is one of the common localization methods which can achieve centimeter-level localization accuracy. In a UWB localization based on TDOA system for UAV swarm, a number of spatially separated receivers (i.e., anchors) capture the UWB signals emitted by the UAV with UWB sensors and estimate TDOAs to locate the UAV (i.e., tag) in an indoor environment.

The basic principle of TDOA algorithm is to firstly measure the arrival time difference between the UAV signal to each known anchor, then multiply the electromagnetic wave propagation speed to obtain the distance difference with which some hyperbola equations are constructed.

Assume there are *A* anchors placed in a 3-D localization zone, the location of the anchor *i* is $x_{i}^{a},i=1,2,...A\left(A\geq 4\right)$, and the location of the UAV is ***x***_*n*_. The UAV sends a UWB signal once in a 3-D indoor area, and all anchors within the UAV localization distance receive the wireless signal. *R*_*i*_ is the distance from the UAV to the anchor *i*, and *R*_*i*_ is defined as:

$$\begin{array}{c} R_{i}=\|x_{i}^{a}-x_{n}\|=\sqrt{{\left(x_{i}^{a}-x_{n}\right)}^{2}+{\left(y_{i}^{a}-y_{n}\right)}^{2}+{\left(z_{i}^{a}-z_{n}\right)}^{2}} \end{array}$$ (4)

*R*_*i*,1_ represents the difference between the distance from the UAV to anchor 1 and to anchor *i*:

$$\begin{array}{c} R_{i,1}=s\tau_{i,1}=R_{i}-R_{1}+sr_{i,1} \end{array}$$ (5)

where *s* stands for the speed of UWB radio wave, *τ*_*i*,1_ stands for the TDOA from the UAV to anchor 1 and anchor *i*, and *r*_*i*,1_ is the additive white gaussian noise (AWGN) with zero-mean and variance *η* = *E*[|*r*_*i*,1_|^2^] = *σ*^2^. Based on Eqs (4) and (5), we can get:

$$\begin{array}{c} R_{i}^{2}-R_{1}^{2}=2\left[X_{i,1}\;Y_{i,1}\;Z_{i,1}\right]{\left[x_{n}\;y_{n}\;z_{n}\right]}^{T}+K_{i}-K_{1} \end{array}$$ (6)

where $X_{i,1}=x_{i}^{a}-x_{1},Y_{i,1}=y_{i}^{a}-y_{1},Z_{i,1}=z_{i}^{a}-z_{1}$, and $K_{i}={x_{i}^{a}}^{2}+{y_{i}^{a}}^{2}+{z_{i}^{a}}^{2}$.

Solving Eq (6) gives the location of the UAV which is not an easy task because the equations involved are nonlinear. The Chan algorithm is one of the methods to tackle this problem.

### Chan algorithm

The Chan algorithm uses the weighted least squares (WLS) method twice to solve the Eq (6), in which the initial nonlinear equations are converted into linear equations with relevant TDOA data firstly, then the first WLS gives an initial solution, and the second WLS makes use of the known constraint between the UAV coordinates and the extra variable, gives an improved location estimate. For more specific calculation results and formula derivation, please refer to [19].

In general, the Chan algorithm is a typical localization algorithm which has high localization accuracy when the TDOA error obeys the Gaussian distribution and the variance of noise is small.

## Proposed algorithms

### Firefly swarm intelligence based cooperative localization

At present, the most commercially available UAV localization method is GPS [35]. This commonly used localization method has high localization accuracy in an outdoor unobstructed environment, but it is susceptible to interference and the signal is unstable in a more complex indoor environment. Therefore, a stable localization method is needed to more accurately locate the UAV indoors.

As analyzed in the last section, the Chan algorithm is a typical localization algorithm which has high localization accuracy when the TDOA error obeys the Gaussian distribution and the variance of noise is small. However, due to the complexity of the indoor environment and the nonlinear problem of localization estimation, the Chan algorithm has a large error when noise power is large.

The TDOA estimation problem is a nonlinear and non-convex optimization problem as well. As a random search algorithm of SI, the FA is simple in principle and easy to implement which is an effective method for solving nonlinear equations. However, the FA is prone to fall into the local optimum solution and premature convergence.

In this paper, we utilize their respective advantages and propose a cooperative localization based on the Chan algorithm and FA to obtain more precise locations of UAVs.

The maximum likelihood method is used to estimate the coordinates of the UAV (*x*_*n*_, *y*_*n*_, *z*_*n*_). We set the TDOA vector is ***τ*** = [*τ*_2,1_, *τ*_3,1_, ⋯, *τ*_*A*,1_]^T^, which obeys the *A*−1 dimensional Gaussian distribution, and the distance mean vector is:

$$\begin{array}{c} \Delta R={\left[r_{2,1},r_{3,1},\cdots ,r_{A,1}\right]}^{T} \end{array}$$ (7)

The actual distance vector **r** from UAV to anchor *i* and the actual distance vector **r**_1_ from UAV to anchor 1 are set as **r** = [*r*_2_, *r*_3_. ⋯,*r*_*A*−1_]^T^ and **r**_1_ = [*r*_1_, *r*_1_. ⋯,*r*_1_]^T^ respectively. According to the previous assumptions, The variables in Δ**R** are independent identically distributed Gaussian random variables with a mean value of *r*_*i*_ − *r*_1_.

Therefore, we have the likelihood function:

$$\begin{array}{c} l={\left(\frac{1}{\sqrt{2\pi }\sigma }\right)}^{A-1}\text{exp}\left(-\frac{1}{2\sigma^{2}}{\left(\Delta R-r+r_{1}\right)}^{T}\left(\Delta R-r+r_{1}\right)\right) \end{array}$$ (8)

where *σ* is the variance of additive white gaussian noise (AWGN). Thus, the estimated location closest to the actual location is equal to the coordinates value that maximizes the likelihood function for the FA:

$$\begin{array}{c} \left(x_{n},y_{n},z_{n}\right)=\text{argmax}\left(-{\left(\Delta R-r+r_{1}\right)}^{T}\left(\Delta R-r+r_{1}\right)\right) \end{array}$$ (9)

We can see this equation is a very complex nonlinear function, and the maximum likelihood estimate can be obtained by searching the coordinates of the UAV corresponding to the minimum value of this function, but the computation of direct search is very high. Therefore, we use the FA to search the optimal solution in the whole potential solution space to determine the location of the UAV, taking advantage of the fact that the FA basically has no restriction on the optimization function [34].

**Fig 2:**
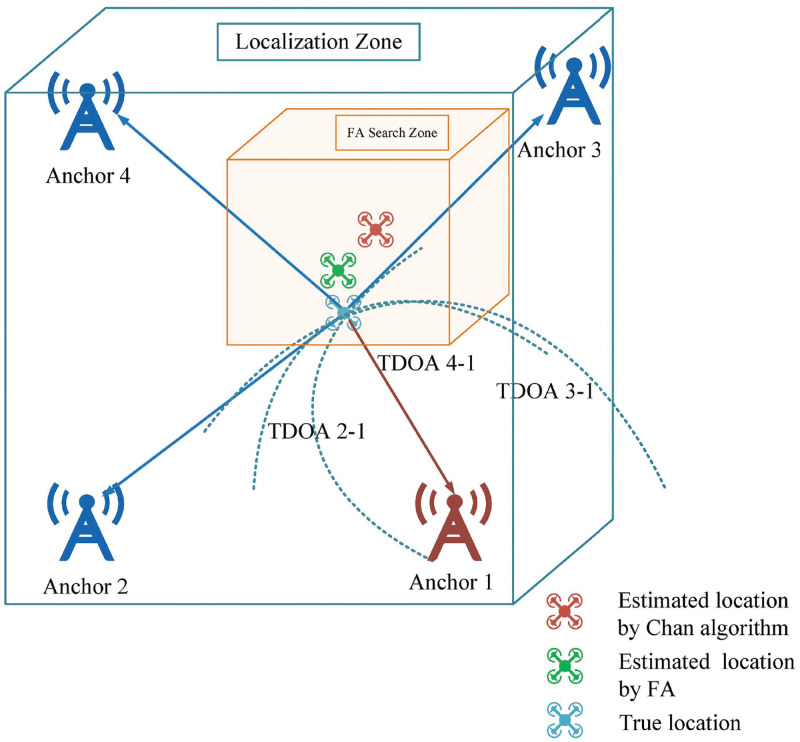
Diagram of the proposed cooperative localization.

**Fig 3:**
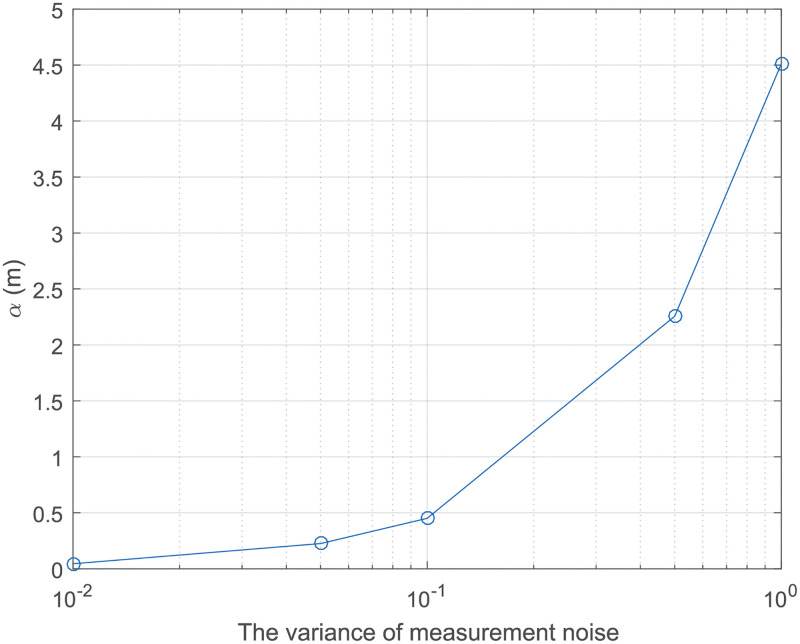
*α* v.s. the variance of measurement noise.

Combining the computational ability of the Chan algorithm and the swarm search ability of the FA, we propose a cooperative localization algorithm as shown in Fig 2. The first solution is obtained by the Chan algorithm, the FA then is used to obtain a more accurate solution in the limited cube search zone centered on the first solution. Since each firefly in the space represents a localization estimation and search zone is narrowed, the probability of the FA falling into the local optimum solution is reduced, the convergence speed is raised, and the localization accuracy is increased. The process of cooperative localization algorithm is described as follows:

1. The first solution $x_{n}^{c}$=($x_{n}^{c}$, $y_{n}^{c}$, $z_{n}^{c}$) is calculated by the Chan algorithm, and the $R_{i,1}^{c}$ from the solution to each anchor is obtained, which is defined as:
  $$\begin{array}{c} \begin{array}{cc} R_{i,1}^{c}= & \sqrt{{\left(x_{i}-x_{n}^{c}\right)}^{2}+{\left(y_{i}-y_{n}^{c}\right)}^{2}+{\left(z_{i}-z_{n}^{c}\right)}^{2}}\\ & -\sqrt{{\left(x_{1}-x_{n}^{c}\right)}^{2}+{\left(y_{1}-y_{n}^{c}\right)}^{2}+{\left(z_{1}-z_{n}^{c}\right)}^{2}} \end{array} \end{array}$$ (10)
  We define the difference between $R_{i1}-R_{i1}^{c}$ for the Chan algorithm as:
  $$\begin{array}{c} \alpha =\sum_{i=2}^{A-1}{\left\|R_{i,1}-R_{i,1}^{c}\right\|}_{2} \end{array}$$ (11)
2. A search zone that is too small may cause the UAV solution to be excluded from the space, while a search zone that is too large will increase the search time and be more likely to fall into a local optimum solution. Based on the change of *α* as shown in Fig 3, we can infer that the distance between the estimated location and the actual location of the UAV is less than *αγ*, where *γ* is the empirical coefficient. Thus, we then set up a cube search zone with side length 2*αγ* centered on the first solution $x_{n}^{c}$ for firefly swarm search, and the TDOA $R_{i,1}^{f}$ from the solution $x_{n}^{f}=(x_{n}^{f}$, $y_{n}^{f}$, $z_{n}^{f}$) obtained by the FA to each anchor is obtained, which is defined as:
  $$\begin{array}{c} \begin{array}{cc} R_{i,1}^{f}= & \sqrt{{\left(x_{i}-x_{n}^{f}\right)}^{2}+{\left(y_{i}-y_{n}^{f}\right)}^{2}+{\left(z_{i}-z_{n}^{f}\right)}^{2}}\\ & -\sqrt{{\left(x_{1}-x_{n}^{f}\right)}^{2}+{\left(y_{1}-y_{n}^{f}\right)}^{2}+{\left(z_{1}-z_{n}^{f}\right)}^{2}} \end{array} \end{array}$$ (12)
  We define the difference between $R_{i,1}-R_{i,1}^{f}$ for the FA as:
  $$\begin{array}{c} \delta =\sum_{i=2}^{A-1}{\left\|R_{i,1}-R_{i,1}^{f}\right\|}_{2} \end{array}$$ (13)
3. When the limited max iterations of the FA are reached, the solution of the FA is the final location of the UAV if *δ* ≤ *α*; whereas, the solution of the Chan algorithm is the final location of the UAV.

**Fig 4:**
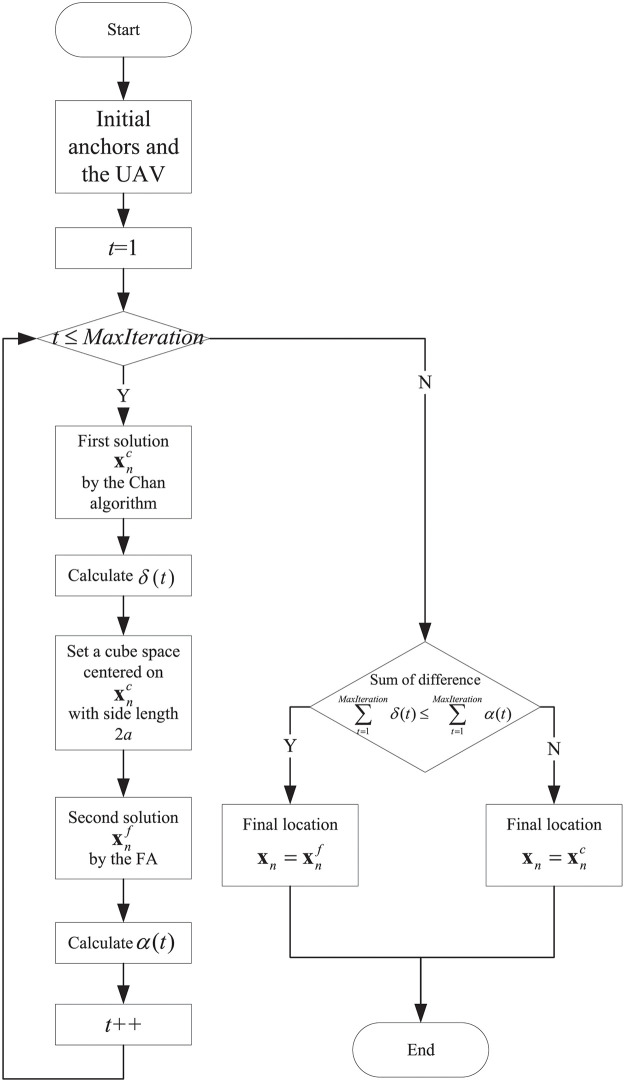
The flowchart of the FSICL algorithm.

The cooperative localization algorithm with *O*(*MaxIteration* * *N*^2^) computational complexity is shown in Algorithm 1, and the flowchart of this algorithm is shown in Fig 4.

**Algorithm 1 FSICL Algorithm**

**Input**: Swarm size *N*, TDOAs ***τ***

**Output**: location ***x***_*n*_

1: /* Initialization Phase 1*/

2: Initialize *A* anchors in the 3-D search zone

3: Initialize a UAV at location ***x***_*n*_

4: /* Computation Phase 1*/

5: **while** (*t*≤*MaxIteration*)

6: Use the Chan algorithm to obtain the first solution $x_{n}^{c}$

7: Calculate *α*(*t*), and set a cube space with side length 2*α* centered on $x_{n}^{c}$

8: /* Initialization Phase 2*/

9: Initialize *N* fireflies in the 2*α* × 2*α* × 2*α* area

10: /* Computation Phase 2*/

11:  **for** *n* = 1: *N*

12:   Calculate fitness function *I*_*n*_ = *l*(***τ***;***x***) using Eq (34)

13:   **for** *m* = 1: *N*

14:    Calculate fitness function *I*_*m*_ = *l*(***τ***;***x***) using Eq (34)

15:    **if** (*I*_*n*_ > *I*_*m*_)

16:     Move firefly *m* towards *n*.

17:    **end if**

18:    Update location and evaluate new light intensity

19:   **end for** *m*

20:  **end for** *n*

21:  Rank the fireflies and find the current brightest $I_{n}^{*}$ at location $x_{n}^{f}$

22:  Calculate *δ*(*t*) by $x_{n}^{f}$

23: **end while**

24: **if** ($\sum_{t=1}^{MaxIteration}\delta (t)\leq \sum_{t=1}^{MaxIteration}\alpha (t)$)

25:  $x_{n}=x_{n}^{f}$

26:  **else if** ($\sum_{t=1}^{MaxIteration}\delta (t)\geq \sum_{t=1}^{MaxIteration}\alpha (t)$)

27:  $x_{n}=x_{n}^{c}$

28: **end if**

### Firefly swarm intelligence based automatic clustering

For a large-scale, high-speed FANET, clustering is one of the effective methods for UAV topology control. The process of CH selection and cluster formation are key steps to stabilize network structure, improve communication reliability, and increase UAV lifetime. In this section, we analyze and describe the proposed FSIAC clustering algorithm, including CH selection and clusters formation.

#### Cluster head selection

Aiming at the characteristics of FANETs, we propose a fitness function consists of four parameters, namely the link survival probability, node degree-difference, average distance, and residual energy for CH selection. In order to obtain the fitness value, the UAV will send the Hello message containing the location, velocity information to their neighbor UAVs within *R*.

• ***Link survival probability***

Based on the highly dynamic topology of the network, the link survival probability is proposed to better describe the mobility of UAVs.

With the help of the accurate location obtained by the FSICL algorithm, the current UAV *n* receives the Hello message containing the location and velocity information sent by its neighbor UAV *m* twice consecutively within the period time *T* from location *b* to location *c*, location *d* is the relative location of *m* to *n*, and we assume that within *T*, the relative moving speed and direction of the UAV remain unchanged.

**Fig 5:**
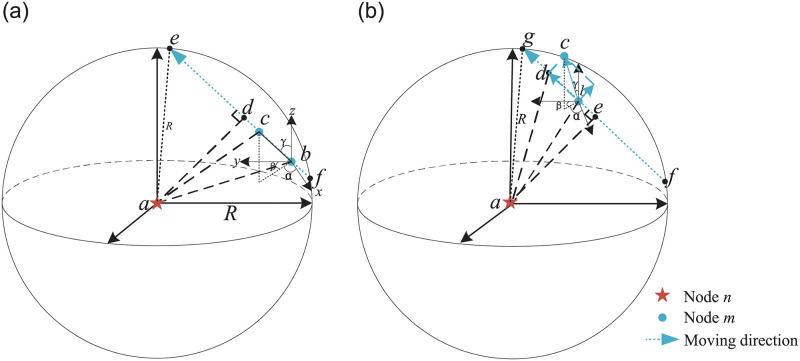
Relative movement models. (a) Approaching model: UAV *m* is approaching UAV *n* from location *b* to location *c*. (b) Leaving model: UAV *m* is leaving UAV *n* from location *b* to location *c*.

Fig 5 shows the relative movement models of the current UAV *n* and its neighbor UAV *m* at speed *v*_*n*_ and *v*_*m*_ respectively. Fig 5(a) represents the UAV *m* is approaching UAV *n* and Fig 5(b) represents the UAV *m* is leaving UAV *n*. UAV *m* sends 2 consecutive Hello messages respectively at the first location *b* and the second location *c*. For two continuous received Hello messages, the coordinates change of UAV *m* in time *T* can be expressed as:

$$\begin{array}{c} \begin{array}{cc} \Delta x_{m} & =x_{m}\left(t+T\right)-x_{m}\left(t\right)\\ & =\sqrt{x_{m}^{2}\left(t+T\right)+y_{m}^{2}\left(t+T\right)+z_{m}^{2}\left(t+T\right)}\\ & \;-\sqrt{x_{m}^{2}\left(t\right)+y_{m}^{2}\left(t\right)+z_{m}^{2}\left(t\right)} \end{array} \end{array}$$ (14)

The movement speed of the UAV *m* can be obtained by Eq (14):

$$\begin{array}{c} v_{m}=\frac{\Delta x_{m}}{T}=\left(v_{m}\text{cos}\alpha ,v_{m}\text{cos}\beta ,v_{m}\text{cos}\gamma \right) \end{array}$$ (15)

where

$$\begin{array}{c} \text{cos}\alpha =\frac{x_{n}\left(t+T\right)-x_{n}\left(t\right))}{\sqrt{\left(\Delta {x_{n}}^{2}-{\left(z_{n}\left(t+T\right)-z_{n}\left(t\right)\right)}^{2}\right)}} \end{array}$$ (16)

$$\begin{array}{c} \text{cos}\beta =\frac{\left(y_{n}\left(t+T\right)-y_{n}\left(t\right)\right)}{\sqrt{\left(\Delta {x_{n}}^{2}-{\left(z_{n}\left(t+T\right)-z_{n}\left(t\right)\right)}^{2}\right)}} \end{array}$$ (17)

$$\begin{array}{c} \text{cos}\gamma =\frac{\left(z_{n}\left(t+T\right)-z_{n}\right)}{\Delta x_{n}} \end{array}$$ (18)

Similarly, the movement speed of the UAV *n* can also be obtained:

$$\begin{array}{c} v_{n}=\frac{\Delta x_{n}}{T}=\left(v_{n}\text{cos}\alpha^{{}^{\prime }},v_{n}\text{cos}\beta^{{}^{\prime }},v_{n}\text{cos}\gamma^{{}^{\prime }}\right) \end{array}$$ (19)

Accordingly, the relative speed between UAV *m* and *n* can be expressed as:

$$\begin{array}{c} \begin{array}{cc} v_{nm} & =v_{m}-v_{n}\\ & =(v_{m}\text{cos}\alpha -v_{n}\text{cos}\alpha^{{}^{\prime }},v_{m}\text{cos}\beta -v_{n}\text{cos}\beta^{{}^{\prime }},\\ & \;v_{m}\text{cos}\gamma -v_{n}\text{cos}\gamma^{{}^{\prime }}) \end{array} \end{array}$$ (20)

Then coordinates of location *e* is:


$$\begin{array}{c} x_{m}^{*}=v_{nm}\cdot T+x_{m} \end{array}$$ (21)

***v***_*nm*_ can be seen as the direction vector of the line *fg*, then we can calculate the lengths of line *de* and *eg* respectively as:

$$\begin{array}{c} \begin{array}{c} \bar{de}=\frac{\left(x_{n}-x_{m}^{*}\right)\cdot v_{nm}}{\left|v_{nm}\right|}\\ \bar{eg}=\sqrt{{\bar{ag}}^{2}-{\bar{ae}}^{2}}=\sqrt{R^{2}-\left({\bar{ad}}^{2}-{\bar{de}}^{2}\right)} \end{array} \end{array}$$ (22)

We define the change of distance difference between UAV *m* and *n* in period *T* as:

$$\begin{array}{c} \Delta d_{nm}=d_{nm}\left(t+T\right)-d_{nm}\left(t\right) \end{array}$$ (23)

In the approaching and leaving models as shown in Fig 5, we can find out Δ*d*_*nm*_ < 0 in the approaching model, while Δ*d*_*nm*_ > 0 in the leaving model.

The link survival probability between UAV *n* and *m* is related to the relative speed and the distance between them. Therefore, the link survival probability can be expressed as:

$$\begin{array}{c} Link_{n}^{m}=\{\begin{array}{c} \begin{array}{c} \frac{\bar{eg}+\bar{ed}}{|v_{nm}|t_{\text{max}}},\;\;\;\Delta d_{nm}<0\\ \frac{\bar{eg}-\bar{ed}}{|v_{nm}|t_{\text{max}}},\;\;\;\Delta d_{nm}>0\\ 1,\;\;\;\;\;\;v_{nm}=0 \end{array} \end{array} \end{array}$$ (24)

where *t*_max_ is the normalization factor which is defined as:

$$\begin{array}{c} t_{\text{max}}=\frac{2R}{v_{nm}} \end{array}$$ (25)

Consequently, the total link survival probability of UAV *n* can be obtained:

$$\begin{array}{c} W_{n}^{1}=\sum_{m\in M_{n}}^{M_{n}}Link_{n}^{m} \end{array}$$ (26)

The bigger the link survival probability, the more stable the network topology, and the lower CHs update frequency. Therefore, UAV *n* with a bigger $W_{n}^{1}$ is more likely to be selected as a CH.

• ***Node degree-difference***

Each UAV acts as a node in the FANET, and due to the limited network bandwidth resources, the number of UAVs in a cluster should be controlled. A large node degree reduces the number of clusters, but it may cause network congestion and affect service quality, while a small node degree causes a serious waste of valuable network bandwidth resource. In the network, the node *n* can transmit Hello message to its neighbors to obtain the node degree, which is defined as:

$$\begin{array}{c} Degree_{n}=\sum_{m\in M_{n}}^{M_{n}}Connectivity_{n}^{m} \end{array}$$ (27)

The optimal number of UAVs in a network is affected by the intra-cluster and inter-cluster communication bandwidth and the number of UAVs in the entire network. According to [36], let *B*_1_ represent the bandwidth of intra-cluster communication, *B*_2_ represent the bandwidth of inter-cluster communication, the optimum number of UAVs in a cluster is:

$$\begin{array}{c} Degree_{ideal}=\frac{B_{2}}{B_{1}}\sqrt{N} \end{array}$$ (28)

The node degree-difference of UAV *n* is the degree of similarity between the actual node number and the theoretical optimal node number, which is defined as:

$$\begin{array}{c} Difference_{n}=\|Degree_{n}-Degree_{ideal}\| \end{array}$$ (29)

The parameter $W_{n}^{2}$ is defined as:

$$\begin{array}{c} W_{n}^{2}=-Difference_{n} \end{array}$$ (30)

UAV *n* with a bigger $W_{n}^{2}$ is more likely to be selected as a CH.

• ***Average distance***

Since the FANET is a dynamic network, and the distance between UAVs is constantly changing. When two UAVs are far apart, the connection between them is unreliable, so the average distance between UAVs is one of the factors that should be considered. The average distance between UAV *n* and all its neighbor UAVs *Distance*_*n*_ as:

$$\begin{array}{c} Distance_{n}=\frac{\sum_{m\in M_{n}}^{M_{n}}d_{nm}}{\left|M_{n}\right|} \end{array}$$ (31)

The parameter $W_{n}^{3}$ is defined as:

$$\begin{array}{c} W_{n}^{3}=-Distance_{n} \end{array}$$ (32)

UAV *n* with a bigger $W_{n}^{3}$ is more likely to be selected as a CH.

• ***Residual energy***

This paper proposes the calculation method of UAV residual energy, considering the influence of node degree on energy consumption when the node acts as a CH and a CM at different speed, making the calculation method more reasonable. Assuming that the initial energy of the UAV *n* is *E*, the residual energy is $W_{n}^{4}$. When the UAV acts as a CM, the energy consumed per node degree per unit time is *e*_1_, and when it acts as a CH, the energy consumed per node degree per unit time is *e*_2_, and when it is hovering, the energy consumed per unit time is *e*_*h*_, then the residual energy of the UAV *n* after a period of time is:

$$\begin{array}{c} \begin{array}{cc} W_{n}^{4}= & E-\sum_{0}^{i}e_{1}Degree_{ni}t_{i}-\sum_{0}^{j}e_{2}Degree_{nj}t_{j}\\ & -e_{h}t_{h}-\sum_{i=0,j=0}^{i,j}G_{n}v_{n}\left(t_{i}+t_{j}\right) \end{array} \end{array}$$ (33)

where *i* is the number of times the UAV *n* acts as a CM, *Degree*_*ni*_ is the node degree of the UAV *n* is acting as a CM for the *i*-th time, *t*_*i*_ is the time during the UAV acts as a CM for the *i*-th time. *j* is the number of times the UAV acts as a CH, *Degree*_*nj*_ is the node degree of the UAV *n* is acting as a CH for the *j*-th time, *t*_*j*_ is the time during the UAV acts as a CH for the *j*-th time, *t*_*h*_ is the hovering time of UAV, *G*_*n*_ is the weight of UAV *n*.

As a special node in the cluster, the CH not only consumes the energy to process the data of CMs uploaded in the cluster, but also undertakes the heavy data transmission task. Compared to the CH, the CM only needs less power to send data to the CH that are closer, not to the GS that may be farther away. Therefore, The higher the residual energy of UAV *n*, the more reasonable it is to be selected as a CH.

• ***Fitness function***

Eventually, based on the preceding discussions, the link survival probability $W_{n}^{1}$, the node degree-difference $W_{n}^{2}$, the average distance $W_{n}^{3}$ and the residual energy parameter $W_{n}^{4}$ of UAV *n* of the UAV can be obtained, and known by all neighbors. Combining these four parameters, a fitness function can be formed as the light intensity *I*_*n*_ in the proposed clustering algorithm and the UAV *n* is selected as a CH with the highest fitness value, which is described as:

$$\begin{array}{c} I_{n}=w_{1}\times W_{n}^{1}+w_{2}\times W_{n}^{2}+w_{3}\times W_{n}^{3}+w_{4}\times W_{n}^{4} \end{array}$$ (34)

where *w*_1_, *w*_4_, *w*_3_, *w*_4_ are the weights of 4 parameters respectively, and *w*_1_ + *w*_2_ + *w*_3_ + *w*_4_ = 1. Their values can be determined according to the task. In this paper, we treat the 4 parameters as equally important, i.e., *w*_1_=*w*_4_=*w*_3_=*w*_4_=0.25.

#### Cluster formation

**Fig 6:**
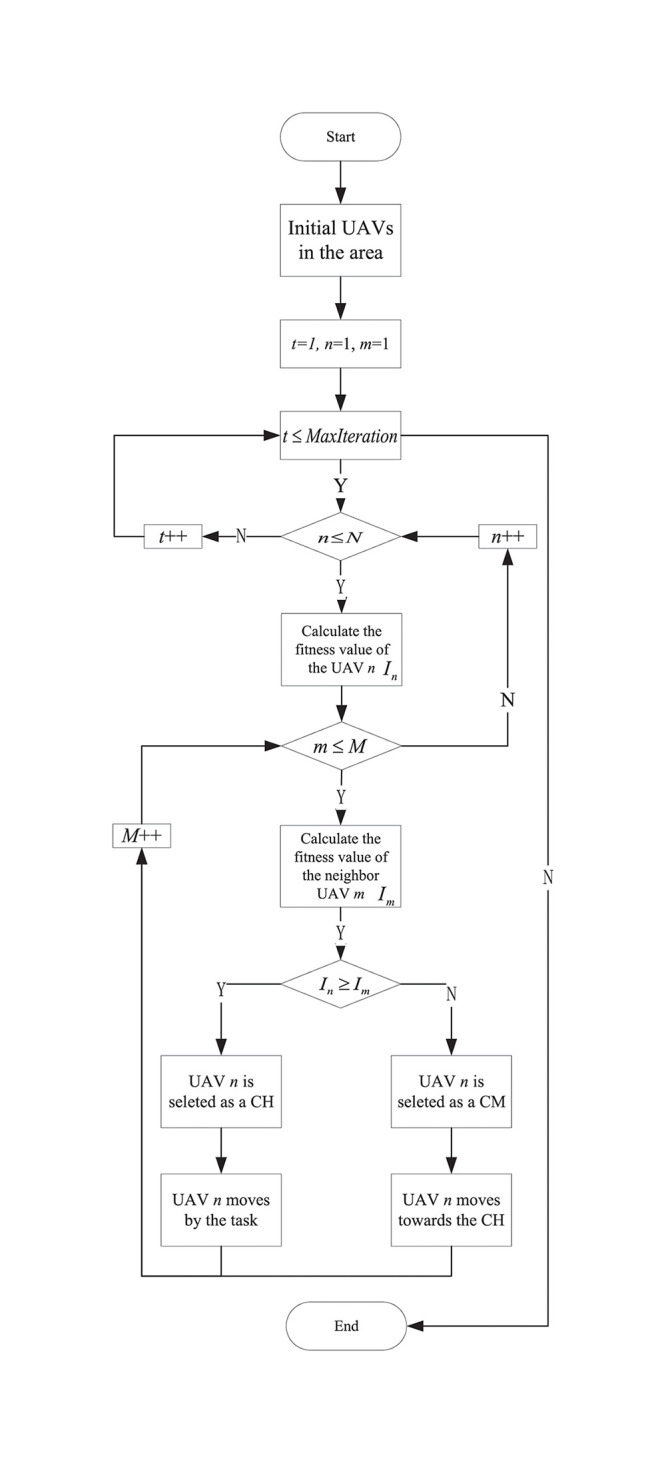
The flowchart of the FSIAC algorithm.

In the FANET, the fast UAV movement makes UAVs to join or leave the clusters frequently, which is more likely to cause greater routing overhead and increase computing load. Cluster formation of FSIAC is performed to add new UAVs, delete UAVs and change the CHs where every UAV is treated as a firefly. A darker UAV tracks a brighter UAV within its field of view, which causes the whole UAV swarm to be automatically divided into several clusters. The light intensity of each UAV updates based on the fitness function, and CH manages the cluster by receiving the location and velocity information of all UAVs within the communication range and constantly updating the topology table. CMs need to adjust their locations spontaneously to track the motion of the CH, thus prolonging the CH holding time and reducing the CH handover rate, improving the clustering stability. Algorithm 2 shows the proposed FSIAC algorithm with *O*(*MaxIteration* * *N* * *M*_*n*_) computational complexity, and the flowchart of this algorithm is shown in Fig 6.

**Algorithm 2 FSIAC Algorithm**

**Input**: Swarm size *N*

**Output**: Clusters *C*

1: /* Initialization Phase*/

2: Initialize *N* UAVs in the search area

3: Assign randomly location for *N* UAVs

4: Assign different moving speed for *N* UAVs

5: /* Computation Phase*/

6: **while** (*t*≤*MaxIteration*)

7:  **for** *n* = 1: *N*

8:   Calculate fitness function *I*_*n*_ using Eq (34)

9:   **for** *m* = 1: *M*_*n*_

10:    Calculate fitness function *I*_*m*_ using Eq (34)

11:    **if** (*I*_*n*_ ≥ *I*_*m*_)

12:    UAV *n* declares itself as a CH through a Hello message and UAV *m* moves towards UAV *n*.

13:    **else**, UAV *m* declares itself as a CH through a Hello message and moves by the task.

14:    **end if**

15:    Update location and evaluate new fitness value

16:   **end for** *m*

17:  **end for** *n*

18: **end while**

## Simulation results

**Fig 7:**
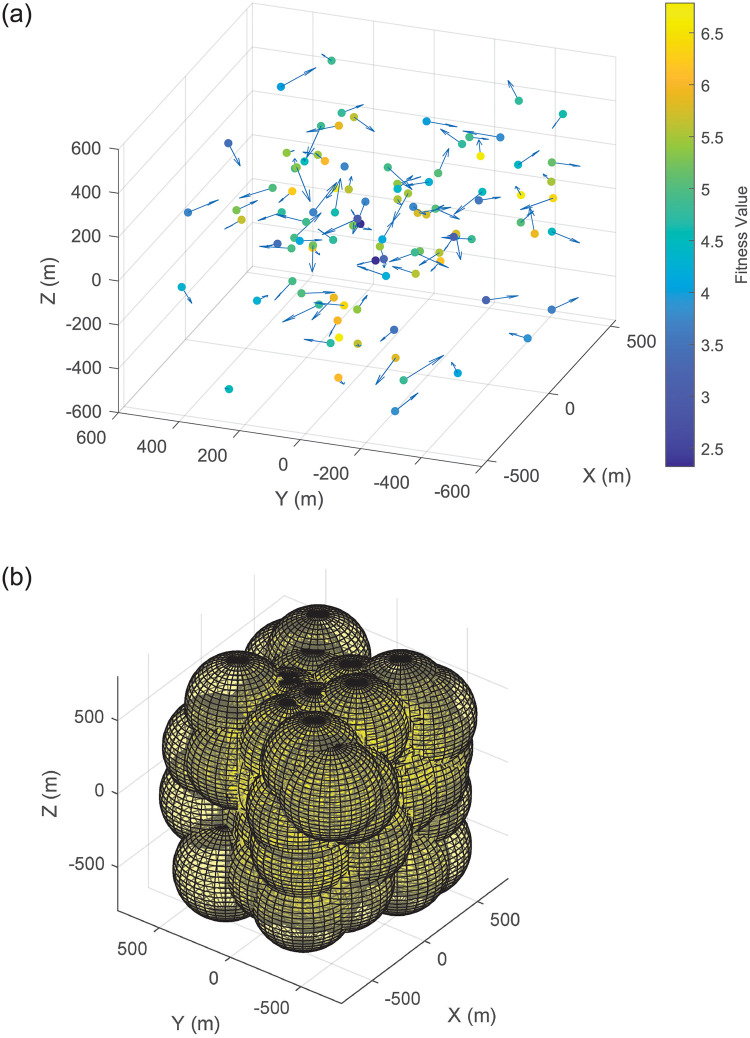
Initial distribution and communication radii of UAVs. (a) Initial locations and moving directions of UAVs (the arrow line represents the moving direction). (b) Initial communication radii of UAVs (the yellow sphere with the UAV as the center represents the communication range of the UAV).

In this section, the performance of the proposed FSICL and FSIAC algorithms are evaluated via simulation in Matlab (R2021b) in a 64-bit computer with a AMD Ryzen R5 3400G processor and 16 GB RAM. Table 1 lists out main simulation parameters. Fig 7 shows the initial distribution of 100 UAVs with moving directions and fitness values and 3-D spherical communication radii of UAVs.

**Table 1:** Simulation parameters.

Parameters	Values
Network area	1000m × 1000m × 1000m
Movement model	Random direction model
Number of UAVs	100
Communication radius *R*	300m
Moving speed	1–4m/s
Initial energy *E*	20J
Randomization *α*	0.3
light absorption coefficient *μ*	0.2
Hello message period *T*	0.1s
The number of fireflies in FSICL	1000
Localization iteraion	1000

### Localization performance

#### Location estimation

**Fig 8:**
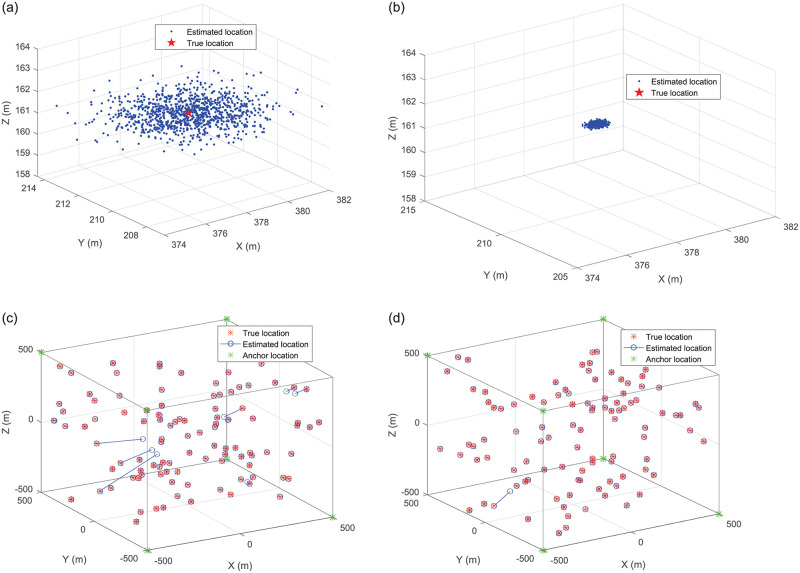
Localization results with 6 anchors when the variance of noise is 1 Watt. (a) 1000 estimated locations of a UAV by the Chan algorithm. (b) 1000 estimated locations of a UAV by FSICL. (c) Average estimated locations of 100 UAVs of 1000 iterations by the Chan algorithm. (d) Average estimated locations of 100 UAVs of 1000 iterations by FSICL.

Fig 8 visually shows localization results with 6 anchors when the variance of noise is 1 Watt. We can see that the 1000 estimated locations by the proposed FSICL are closer to the actual location of the UAV than the Chan algorithm as shown in Fig 8(a) and 8(b). We further demonstrate the localization result in terms of estimated locations of UAVs versus actual locations of the UAVs as shown in Fig 8(c) and 8(d). We can see that there are five UAVs with large localization error by the Chan algorithm while there is only one UAV with large localization error by FSICL. In this case, the proposed FSICL algorithm has higher localization accuracy than the Chan algorithm.

#### Convergence

**Fig 9:**
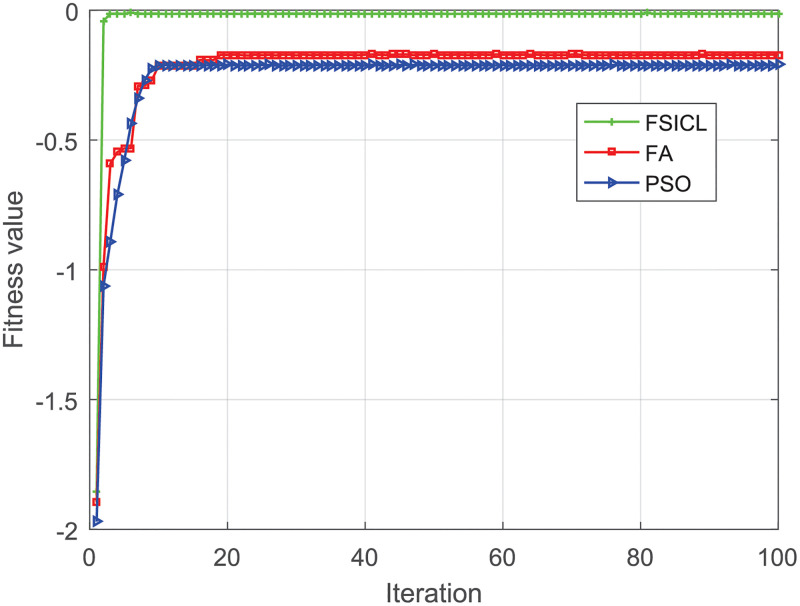
Fitness value v.s. iteration.

Fig 9 shows the proposed FSICL converges to a bigger fitness value faster the Chan algorithm and PSO, which means the proposed FSICL uses less time to get a more accurate location of the UAV than the Chan algorithm and PSO. This is because the FSICL works in the smaller space around the first estimated location by the Chan algorithm, which helps to narrow search zone and search time of the FA. It should be noted that, the fitness values obtained by the FA and PSO are smaller than that obtained by the FSICL, which can be explained as that both the FA and PSO algorithms converge to their local optimums. Furthermore, the value obtained by the PSO is smaller than that obtained by the FA, which represents the FA is less likely to fall into the local optimum than the PSO due to its advantage of finding both the global optimum and the local optimums.

#### Root mean square error

**Fig 10:**
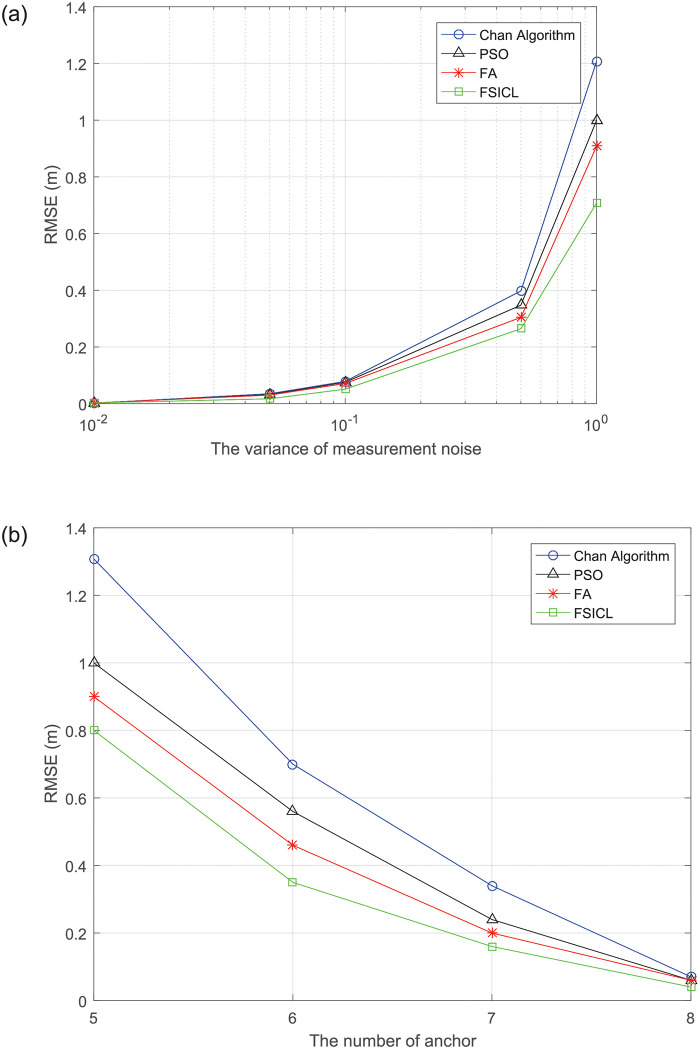
Average RMSE results after 1000 iterations. (a) RMSE v.s. the variance of noise. (b) RMSE v.s. the number of anchor.

Fig 10(a) shows the average root mean square error (RMSE) varies with the variance of measurement noise. RMSE is a measure of the deviation between the estimated location and actual location. The lower the RMSE, the higher the localization accuracy. We can see that RMSEs of all localization algorithms increase with the increase of measurement noise. In these algorithms, the proposed FSICL achieves the lowest RMSE. This is because the proposed FSICL algorithm directly searches for the solution of Eq (9) from the maximum likelihood method, and the noise has little effect on it. Therefore, the proposed algorithm has better performance in terms of noise resistance. Fig 10(b) presents the RMSE with varying the number of anchors. We can see RMSEs of all algorithms decrease with the increase of the number of anchors, this is due to the increase in the number of anchors, which increases more redundant information and improves the localization performance accordingly. In addition, since the FSICL algorithm combines the computing ability of the Chan algorithm and swarm search ability of the FA to locate UAVs, the proposed FSICL achieves the lowest RMSE compared with the other three algorithms.

### Clustering performance

#### Connectivity

**Fig 11:**
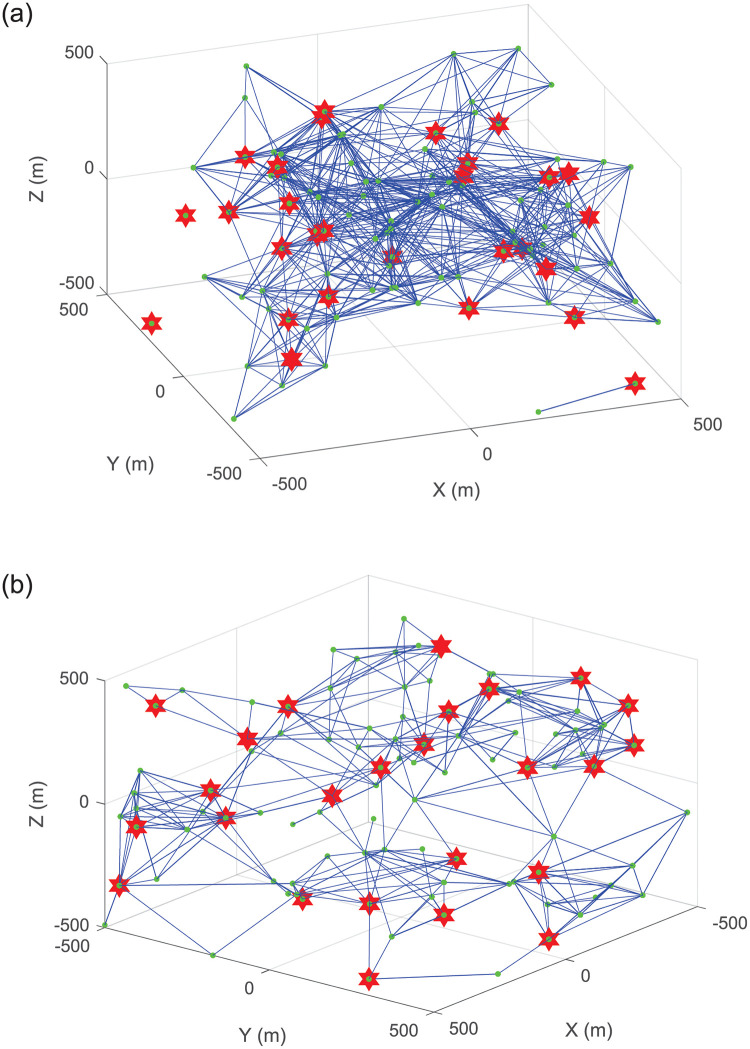
Connectivity of UAVs after different iterations. (a) First itration. (b) Tenth itration.

Fig 11(a) and 11(b) intuitively display the connectivity of UAVs at the speed of 1m/s after different iterations. We can see that the distribution of CMs after ten iterations is more balanced than the initial distribution and the communication burden of each CH is allocated more evenly. This is because we consider the node degree when selecting the CHs. The UAV with an appropriate number of node degree is more likely to be selected a CH, which leads to a certain distance between CHs. In addition, CMs will adjust their locations on the basis of their fitness values to automatically track the motion of CHs, which makes the distribution of UAVs more evenly.

#### Stability

**Fig 12:**
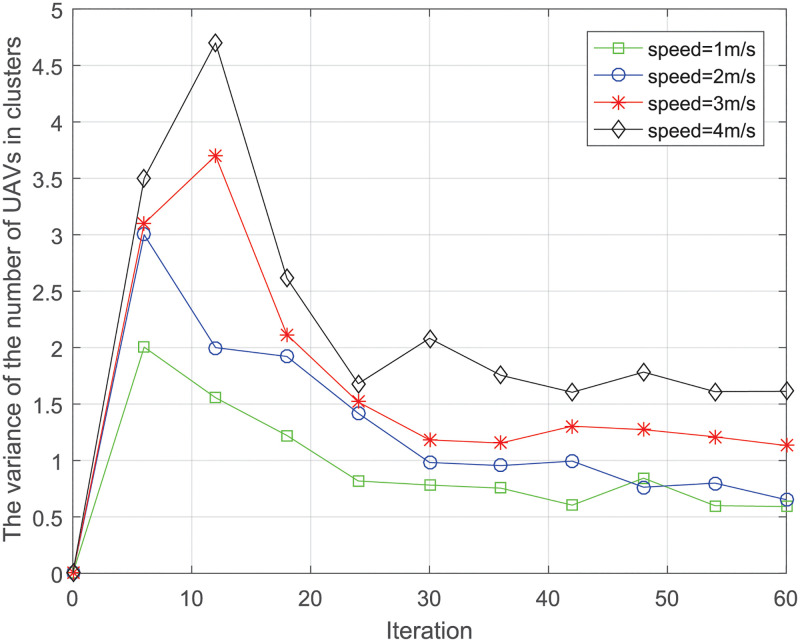
The variance of the number of UAVs in clusters v.s. iteration.

Fig 12 shows the variance of the number of UAVs in clusters after different iterations with different speeds. The variance of clusters at time *t* is defined as:

$$\begin{array}{c} Var\left(t\right)=\frac{\sum_{n=1}^{N}{\left(Degree_{n}\left(t\right)-\frac{1}{N}\cdot \sum_{n=1}^{N}Degree_{n}\left(t-1\right)\right)}^{2}}{N} \end{array}$$ (35)

The smaller the variance, the more stable the number of CMs are in the cluster. We can see that variance is greater when speed is faster. This is because the high mobility of network UAVs leads to the faster updating of network topology and disconnection of communication links, resulting in more frequent cluster updates. The faster the speed, the more time it takes to achieve lower variance. When the moving speed of UAV is 1m/s, the swarm can stabilize faster than the other speeds, the faster the communication routing is stabilized.

**Fig 13:**
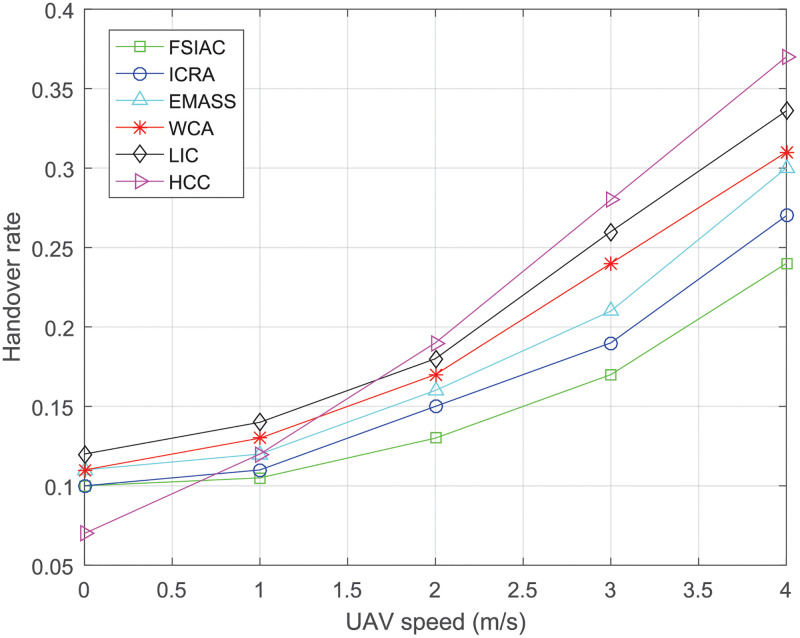
The handover rates of the clusters v.s. UAV moving speed.

Fig 13 reflects the influence of the speed of UAV on the handover rate of CHs in the swarm. The handover rate of CHs is defined as the number of the CHs update in unit time divided by the total number of the network UAVs. With the continuous increase of the moving speeds of UAVs in the network, the handover rate is on the rise. This is because the high mobility of network UAVs will lead to the faster updating of network topology and communication links. Since the proposed FSIAC algorithm comprehensively considers the firefly inspired movement mechanism that CMs are willing to track the CH in the cluster, the handover rate of the proposed FSIAC algorithm is lower than the other five clustering algorithms, which achieves the best stability of CHs.

#### LET

**Fig 14:**
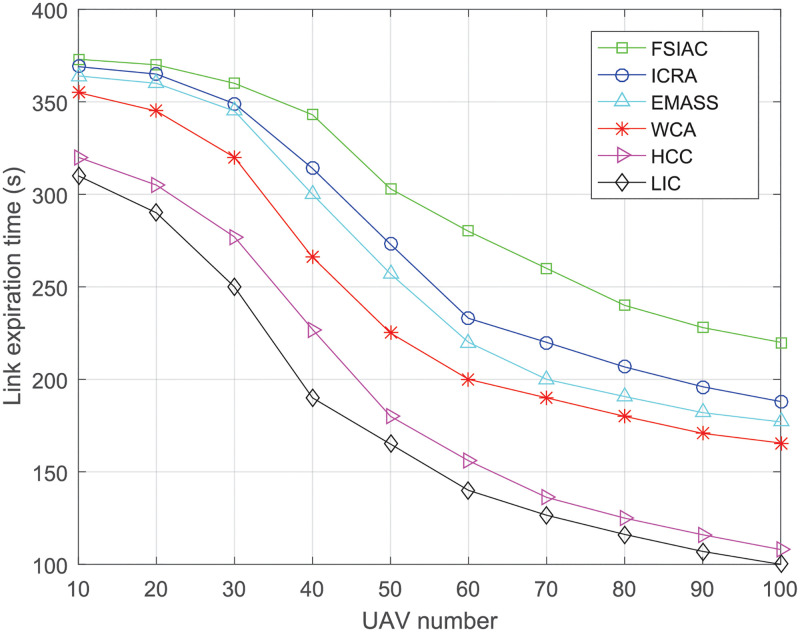
Link expiration time of the clusters v.s. UAV number.

Fig 14 shows the average LET of different clustering algorithms when moving speeds of UAVs are 1m/s. The LET is the average of the time from the connection of the link to the disconnection of the link between the CH and the CMs. The longer the LET, the more stable the intra-cluster communication. We can see from this figure that with the increase of the UAV number, the average LET of the above five algorithms decreases. This is because the network topology changes are more and more frequent due to the increase of the moving UAVs, and the average LET general decreases. With the change of the UAV number, the proposed FSIAC algorithm has the longest LET compared with the LIC, HCC, WCA, EMASS and ICRA algorithms. This is because the proposed FSIAC algorithm considers the link survival probability in fitness function for CH selection, and CMs will adjust their locations to automatically track the CH, which makes the LET longer and intra-communication more stable.

#### Node lifetime

**Fig 15:**
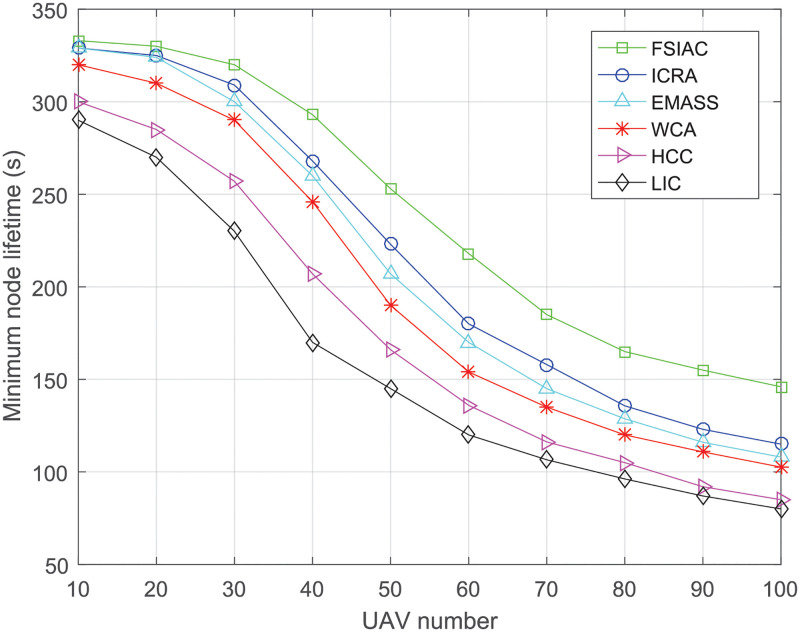
Minimum node lifetime v.s. UAV number.

The node lifetime is defined as the time for a node from being active to inactive, and each UAV is regard as a node in the network. When a clustering scheme executes, the fitness values of CHs and CMs decrease over time and the node is no longer active after it runs out of battery power. As indicated in Fig 15, the minimum node lifetime decreases when more UAVs are introduced to the network. This is because when the UAV swarm is larger, the changes of the network topology become more frequent, which increases the cost of maintaining the topology and energy consumption of the CHs, thus shortening the lifetime of the UAV swarm. Regardless of the increase or decrease of UAV number, our proposed FSIAC algorithm achieves the longest node lifetime compared with the other five algorithms. This is because we consider a more reasonable fitness function to enable timely switching between CHs and CMs, leading to the extension of the entire network lifetime.

### Summary

The summary and comparison of the proposed FSICL and FSIAC algorithms with the other algorithms are listed in Tables 2 and 3.

**Table 2:** Performances of different localization algorithms.

Algorithms	Convergence speed	Accuracy
Chan algorithm		lowest
PSO	medium	low
FA	slowest	high
FSICL	fastest	highest

**Table 3:** Performances of different clustering algorithms.

Algorithms	Stability of clusters	LET	Node lifetime
LIC	low	shortest	shortest
HCC	lowest	short	short
WCA	medium low	medium short	medium short
EMASS	medium high	medium long	medium long
ICAR	high	long	long
FSIAC	highest	longest	longest

We can see that the proposed FSICL algorithm achieves the fastest convergence speed and highest localization accuracy, and the proposed FSIAC algorithm achieves the highest stability of clusters, longest LET and longest node lifetime.

## Conclusion

The FANET is a special application of the MANET in the field of UAV. It not only has the characteristics of centerless and easy to deploy, but also faces the more severe problem of high-speed mobile UAVs, which brings instability to the topology and communication. In this paper, we propose novel FSICL and FSIAC algorithms for FANETs that use the FA to locate UAVs, complete CH selection and cluster formation. The FSICL first combines the FA and Chan algorithm to cooperative locate UAVs based on UWB TDOA technique, which narrows the search zone of the FA based on the initial solution obtained by the Chan algorithm. Then the FSIAC algorithm uses a more appropriate objective function which consists of link survival probability, node degree-difference, average distance and residual energy, and take it as the light intensity of a firefly. The FA finally is used again for CH selection and cluster formation, enabling UAVs to spontaneously cluster around their CHs by tracking them.

The proposed FSICL has been tested in terms of location estimation, convergence, and RMSE of localization. Meanwhile, the proposed FSIAC has been tested in terms of distribution of UAVs, stability of clusters, intra-cluster communication, and node lifetime. The results show the proposed FSICL algorithm effectively achieves the higher localization accuracy in less time, and with the help of the FSICL, the proposed FSIAC algorithm achieves higher stability of clusters, longer LET and longer node lifetime, all of which improve communication performance of the FANET. With the salient performance and technical merits, the proposed FSICL and FSIAC algorithms may serve as practical solutions for indoor FANETs.

With the high localization accuracy of the FSICL algorithm, the verifications of indoor UAVs applications can be well supported by only carrying UWB devices, but it is difficult to deploy UWB anchors in actual tasks. Therefore, in the future, we will study semantics-aware visual localization and the engineering design of the Hello message to realize the real application of the proposed FSIAC algorithm.

## Supporting information

S1 File (RAR)
